# Smoking Cessation, Weight Gain, and Risk for Type 2 Diabetes: A Prospective Study

**DOI:** 10.3389/ijph.2022.1604654

**Published:** 2022-04-14

**Authors:** Lin Wu, Xiaowen Wang, Jia-Yi Dong, Yan-Ting Zhao, Hongqiang Lou

**Affiliations:** ^1^ Department of Medical College, Jinhua Polytechnic, JinHua, China; ^2^ Public Health, Department of Social Medicine, Osaka University Graduate School of Medicine, Osaka, Japan; ^3^ Chengdu Center for Disease and Prevention, Chengdu, China

**Keywords:** smoking cessation, diabetes, weight gain, prospective study, Chinese

## Abstract

**Objectives:** To examine the association between smoking cessation and risk of type 2 diabetes with emphasis on post-cessation weight gain.

**Methods:** In total, 8,951 participants from the China Health and Retirement Longitudinal Study at the baseline (2011) were included. Diabetes incidence was accessed at the third survey (2015). Current smokers were treated as the reference and odds ratios (OR) of type 2 diabetes for never smokers, recent, and long-term quitters were computed using multivariable logistic regression. Stratified analysis was further conducted by weight gain after smoking cessation.

**Results:** There were 712 cases of type 2 diabetes identified. Compared with current smokers, the fully multivariable-adjusted ORs were 1.55 (1.02, 2.36) for recent quitters, 0.88 (0.61, 1.28) for long-term quitters, and 0.75 (0.59, 0.95) for never smokers. Stratified analysis showed recent quitters with weight gain of ≥2.0 kg had a significantly higher odds of type 2 diabetes [2.25 (1.02, 4.95)].

**Conclusion:** The present study of the Chinese population suggested recent quitters with weight gain of ≥2.0 kg, compared with current smokers, had a significantly increased odds of type 2 diabetes.

## Introduction

Cigarette smoking significantly increases the risk of various diseases [1]. Quitting smoking has been shown to be associated with a decreased risk [2]. However, quitting smoking is usually associated with a weight gain during the subsequent few years [3, 4], which raised the concern that weight gain may attenuate the benefits on health. Our recent work examining the risk of CVD in relation to smoking cessation provided evidence that smoking cessation, compared with continuous smoking, was associated with a lower risk of CHD, stroke, total CVD, and all-cause mortality, which was independent of post-cessation weight gain [5].

On the other hand, previous cohort studies showed that smoking quitters, compared with never smokers, had an increased risk of developing type 2 diabetes. The risk was substantially high in recent quitters (<5 years) and decreased as quitting years increased [6–8]. A recent US cohort study using current smokers as the reference found that the recent quitters with post-cessation weight gain greater than 5.0 kg had a 36% increase in the risk of type 2 diabetes, while recent quitters with no weight gain did not have a significantly increased risk [9]. Post-cessation weight gain was therefore considered to directly contribute to the increased risk. On the contrary, a study conducted in Australia reported that weight gain after smoking cessation did not significantly increase the risk of type 2 diabetes [10]. However, a recent Korean study provided evidence showing that smoking cessation without subsequent weight gain was associated with a reduced risk of developing type 2 diabetes, but weight gain could attenuate this reduced risk [11]. However, there was no study conducted among Chinese populations. Given the limited and inconsistent evidence at present, we, therefore, aimed to examine the association between smoking cessation and risk of type 2 diabetes among a Chinese population with emphasis on post-cessation weight gain, using current smoking as the reference.

## Methods

### Participants

**FIGURE 1:**
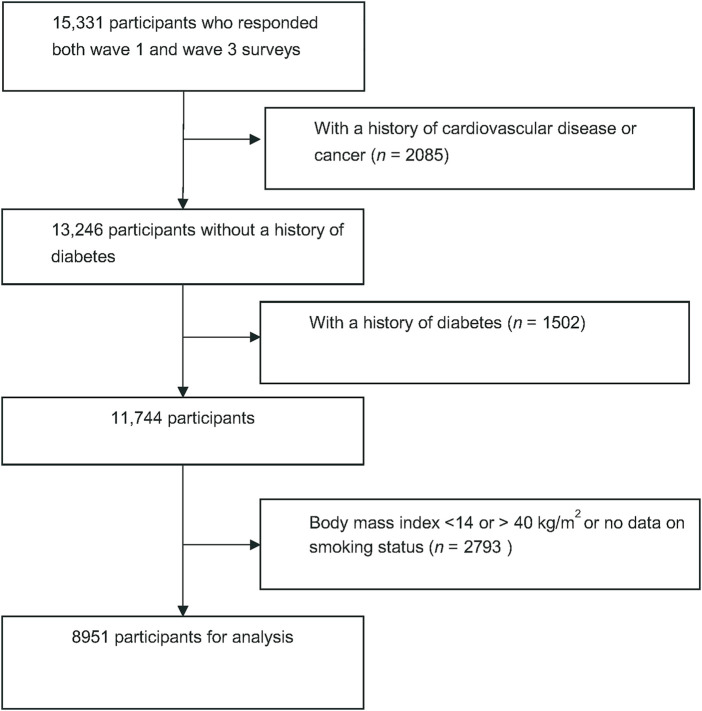
Flow chart of participant selection (JinHua, China. 2022).

The China Health and Retirement Longitudinal Study (CHARLS) is an ongoing and nationally representative longitudinal study that enrolled Chinese residents over 45 years from 450 communities. The details regarding study design, setting, and data collection have been previously described [12]. In brief, a total of 17,708 respondents were enrolled in the baseline survey of 2011–2012 and followed up every 2 years. We first excluded people who reported a history of heart disease, stroke, or cancer at baseline. People with a history of diabetes at baseline diagnosed by doctors, fasting blood glucose ≥126 mg/dl, or HbA1c ≥ 6.5% were excluded. We further excluded people with an extreme body mass index (BMI) (i.e., <14 or >40 kg/m^2^). Ultimately, a total of 8,951 participants were eligible for the final analysis (Figure 1).

CHARLS has obtained Ethics approval from the Biomedical Ethics Review Committee of Peking University (IRB00001052-11015). All the participants have completed a written informed consent.

### Assessment of Smoking Status

In the first and 2-year surveys, each participant was first asked whether they have ever smoked cigarettes. Those who answered yes were further asked whether they still smoked or completely quitted. Former smokers were also asked when they completely quitted smoking. According to the self-reported smoking status in the two surveys, we categorized the participants into four groups. Current smokers were defined as those reporting that they were currently smoking in both surveys. Former smokers who quitted smoking within 2 years were treated as recent quitters and those who quitted smoking longer than 2 years were treated as long-term quitters. Never smokers were defined as those reporting that they never smoked in both surveys.

### Assessment of Weight Change

We focused on short-term weight gain given that considerable weight gain has been reported during the first few years after smoking cessation. Therefore, the baseline assessment of weight change was according to the baseline survey in 2011 and the second survey in 2013. We further categorized the recent quitters into two groups according to the weight change, which was measured as the difference in body weight (kg) in both surveys. Body weight was measured by trained staff in the physical examinations.

### Assessment of Diabetes

Diabetes incidence was determined if one or more of the following criteria was met: 1) reporting a history of diabetes diagnosed by doctors at the third survey in 2015, 2) fasting blood glucose ≥126 mg/dl, or 3) HbA1c ≥ 6.5%. Blood test was not performed in the second survey, and the results of blood test in the third survey were therefore used for assessment of diabetes cases. Venous blood samples were measured for fasting blood glucose and HbA1c by KingMed laboratory.

### Covariates

Data on demographic features, socioeconomic status, personal history of chronic diseases, mental health, physical function, and lifestyles were obtained from a questionnaire. The BMI was calculated as weight (kg)/height (m)^2^. The history of hypertension and dyslipidemia were identified by self-report of doctor diagnosis. The depressive symptoms were accessed using the 10-item Center for Epidemiologic Studies Depression Scale, with a score of 10 or higher was defined as having depressive symptoms [13].

### Statistical Analysis

Participants were divided into four groups according to self-reported smoking status in the baseline survey: never smokers, current smokers, recent quitters, and long-term quitters. Distributions according to smoking status were calculated using the Generalized Linear Model (GLM). Current smokers were treated as the reference group and the odds ratios of type 2 diabetes for never smokers, recent, and long-term quitters were computed using the multivariable logistic regression. Model 1 adjusted for age only. Model 2 further adjusted for sex, education level (primary school or lower, middle school, high school, college or higher), study area (urban or rural area), depressive symptoms (yes or no), marital status (married, divorced, widow, or single), history of hypertension (yes or no), history of hyperlipidemia (yes or no), vigorous activity (yes or no), and drinking status (never drinking, former drinking, less than once per month, and more than once per month). Model 3 further adjusted for baseline BMI (quintile). To examine whether weight change after smoking cessation modified the ORs among recent quitters, we further conducted a subgroup analysis by weight change between the baseline survey in 2011 and the second survey in 2013 (≥2.0 kg or <2.0 kg). We used 2.0 kg as the cut-off point as it has been shown that the average weight gain after smoking cessation was around 2.0 kg among east Asians [14, 15]. All analyses were performed with SAS software version 9.4. A *p* value <0.05 was considered statistically significant.

## Results

Table 1, presents the baseline characteristics of participants according to smoking status in 2011. Compared with current smokers, recent and long-term quitters were more likely to have a higher BMI, have a higher level of education, live in urban areas, have a history of hypertension and dyslipidemia but less likely to be current drinkers and have vigorous activity every day. Never smokers were more likely to be women, have a higher BMI, live in the urban area, have a history of hypertension and dyslipidemia but less likely to have a higher level of education, to be smokers, and to have vigorous activity every day.

**TABLE 1:** Baseline characteristics of Chinese men and women by smoking status (JinHua, China. 2022).

	Current smokers	Recent quitters	Long-term quitters	Never smokers
No. of participants	2,922	247	446	5,336
Age, year	58.8 (9.5)	59.5 (8.9)	62.3 (9.2)	58.3 (9.5)
BMI, kg/m^2^	22.2 (3.5)	22.9 (3.1)	23.8 (3.5)	23.5 (3.4)
Overweight, %	16.2	21.2	26.7	27.5
Obesity	1.7	2.8	4.2	4.8
Waist circumference, cm	81.9	83.7	86	83.9
Men, %	91.1	90.3	87.4	20.6
Higher school or higher education, %	11.2	14.6	12.3	8.4
Married, %	89.6	91.2	90.8	86.6
Living in urban area, %	29.6	32.1	35.7	34
History of hypertension, %	14.7	20.2	20.4	18.8
Dyslipidemia, %	4.4	5.7	8.6	5.8
Current drinker, %	58.7	57.3	51.3	19.1
Moderate activity every day, %	46.7	43.6	39.4	47
Vigorous activity every day, %	33.3	30.7	16.9	22

All values were means (SD) or percentage. Overweight: 25 < BMI< 29.9; Obesity: BMI ≥30.

We identified 712 cases of type 2 diabetes among 8,951 participants based on self-report and blood tests at the third survey in 2015 as shown in Table 2. Compared with current smokers, the age-adjusted ORs of type 2 diabetes were 1.71 (1.13, 2.58) for recent quitters, 1.14 (0.79, 1.64) for long-term quitters, and 1.15 (0.97, 1.37) for never smokers. After adjustment for covariates in model 2, the ORs were somewhat attenuated. After further adjustment for baseline BMI, the ORs were 1.55 (1.02, 2.36) for recent quitters, 0.88 (0.61, 1.28) for long-term quitters, and 0.75 (0.59, 0.95) for never smokers.

**TABLE 2:** Association between smoking cessation and risk of type 2 diabetes (JinHua, China. 2022).

	Cases/participants	Model 1	Model 2	Model 3
Current smokers	210/2,922	1.0	1.0	1.0
Never smokers	436/5,336	1.15 (0.97, 1.37)	0.85 (0.67, 1.08)	0.75 (0.59, 0.95)
Recent quitters	29/247	1.71 (1.13, 2.58)	1.63 (1.08, 2.48)	1.55 (1.02, 2.36)
Weight gain < 2.0 kg	21/196	1.52 (0.94, 2.44)	1.48 (0.92, 2.40)	1.40 (0.86, 2.26)
Weight gain ≥ 2.0 kg	8/51	2.51 (1.16, 5.42)	2.21 (1.01, 4.82)	2.25 (1.02, 4.95)
Long-term quitters	37/446	1.14 (0.79, 1.64)	1.04 (0.72, 1.51)	0.88 (0.61, 1.28)

Model 1: adjusted for age.

Model 2: further adjusted for sex, education level (primary school or lower, middle school, high school, college or higher), study area (urban or rural area), depressive symptoms (yes or no), marital status (married, divorced, widow, or single), history of hypertension (yes or no), history of hyperlipidemia (yes or no), vigorous activity (yes or no), and drinking status (never drinking, former drinking, less than once per month, and more than once per month).

Model 3: Model 2 + baseline BMI.

For recent quitters, we further conducted stratified analysis by weight gain (≥2.0 kg or <2.0 kg) during the first 2 years of follow-up. Compared with current smokers, recent quitters with weight gain of ≥2.0 kg had a significant high odds of type 2 diabetes [fully-adjusted OR = 2.25 (1.02, 4.95)], while recent quitter with weight gain of <2.0 kg did not experience a significantly increased odds [fully-adjusted OR = 1.40 (0.86, 2.26)].

## Discussion

The present study examined the post-cessation risk of developing type 2 diabetes among the Chinese population. Compared with current smokers, recent quitters (former smokers who quitted smoking within 2 years) had a significantly increased odds of developing type 2 diabetes while long-term quitters (those who quitted smoking longer than 2 years) did not experience a significantly increased odds. Further stratified analysis by post-cessation weight gain among recent quitters revealed a significantly higher odds of type 2 diabetes among recent quitters with weight gain of ≥2.0 kg but not among recent quitters with weight gain of <2.0 kg.

The main finding of our study that weight gain after smoking cessation played an important role in the development of type 2 diabetes was consistent with that from a previous study involving three cohorts in the United States, which found that compared with current smokers, the hazard ratios (HR) were 1.36 (1.16, 1.58) among recent quitters with weight gain of 0.1–5.0 kg, but 1.08 (0.93, 1.26) among those without weight gain. Weight gain could potentially modify the association between smoking cessation and the risk of type 2 diabetes [9]. Nevertheless, data from a national Australian household survey indicated that quitters, either weight gain or not, did not significantly increase the risk of type 2 diabetes in comparison with current smokers [HRs = 0.75 (0.35, 1.59) and 0.72 (0.41, 1.27), respectively] [10]. The discrepancy may relate to the characteristics of study participants where the subjects of our study and the US study were middle-aged and older people, while the sample of Australian adults was aged 18 years or older. The older might be at a higher risk of developing cardiometabolic diseases due to age-related glucose dysregulation [16]. On the contrary, a nationally representative database of nearly 100,000 Korean participants aged over 18 years has demonstrated that smoking cessation with no weight gain was associated with a 14% reduced risk of type 2 diabetes [HR = 0.86 (0.80, 0.93)] [11]. The discrepant findings might be explained by the different ethnic groups, the magnitude of weight gain, the selection of confounders, or the length of follow-up. Further large-scale studies with different ethnic populations are warranted to assess the association between post-cessation weight gain and the risk of type 2 diabetes.

Our study suggested that recent smoking quitters who gain substantial weight after quitting were at an increased risk for type 2 diabetes than sustained smokers. Possible mechanisms underlying our findings may be related to the joint chronic effects of smoking and weight gain. There was still deterioration of glucose metabolism changes and insulin resistance after smoking abstinence than baseline long-term smokers, and these alterations might be associated with or contribute to weight gain after smoking cessation [17].

Evidence from a previous observational study also supported that decreasing 10 cigarettes/d in men had a detrimental effect on insulin (7%), glucose (0.11 mmol/L), triglycerides (8%), waist circumference (0.97 cm), and BMI (0.31 kg/m^2^) increases over 3 years [18]. On the other hand, previous studies suggested that body weight variability was an independent risk factor for diabetes [19, 20]. Pani et al. reported that every 1-pound (0.4 kg) increase in weight was associated with a 2% increased odds of type 2 diabetes progression [21]. A cohort study in Finnish male smokers indicated that compared with those of weight change <4.0 kg, weight gain and fluctuation were associated with higher risk for type 2 diabetes for a weight gain of at least ≥4.0 kg, with multivariate RR = 1.77 (1.44, 2.17) [22]. Data from a Korean population aged 30–59 years suggested that the HR (95% CI) for incident diabetes for those of weight gain ≥2.1 kg compared to the stable weight group was 1.24 (1.02, 1.49) over a 5-year follow-up [23]. Weight gain may lead to the disruption of metabolism, such as decreased glucose tolerance [24] and insulin resistance [25], which might amplify or prolong the deterioration of glucose metabolism caused by smoking cessation.

Numerous prior studies have found smoking cessation was associated with a higher risk of type 2 diabetes compared with non-smoking [6–8, 26–28]. For example, a large meta-analysis indicated that compared with never smokers, the pooled relative risk of type 2 diabetes was 1.54 (1.36, 174) for new quitters within 5 years, 1.18 (1.07, 1.29) for quitters of 5–9 years, and 1.11 (1.02, 1.20) for quitters at least 10 years [8]. Our study added evidence on the health issues of smoking cessation rather than smoking with regard to the risk of type 2 diabetes by using current smokers as the reference group. This is the first study conducted in a nationally representative Chinese population and the results from our study highlight the importance of weight management for smoking cessation in the prevention of type 2 diabetes among the Chinese population.

Several limitations of this study must be addressed. First, information on smoking status was gathered by self-reported questionnaires, which might be subject to recall bias. Also, the smoking status might change during the follow-up, which was very likely to have biased the association toward the null. However, less than 10% of the recent or long-term quitters restarted smoking in the first 2 years. Second, the length of follow-up could be relatively short to observe the outcome of chronic diseases, probably resulting in the underestimation of the associations. Third, reverse causation might occur because participants who quitted smoking were more likely to have certain clinical symptoms or a high level of exposure to tobacco. It is suggested that the reasons for smoking quitting should be collected in detail in future studies. Fourth, this study was conducted in the Chinese population, thus the generalizability of our findings may be limited. Moreover, pharmacotherapy treatment for smoking cessation was not considered, which may theoretically decrease post-cessation weight gain. Finally, residual confounding, including measurement errors of covariates or other unmeasured risk factors (e.g., occurrence of diabetes in family history or gestational diabetes in personal history), remained alternative explanations for the observed associations.

In conclusion, the present study of the Chinese population suggested that recent quitters with weight gain of ≥2.0 kg, compared with current smokers, had a significantly increased odds of type 2 diabetes. Our findings revealed that weight management after smoking cessation was of great importance for recent quitters in the prevention of type 2 diabetes.

## Data Availability

The data are publicly available which can be downloaded at http://charls.pku.edu.cn/index/zh-cn.html.

## References

[1] Collaborators GBDT. Smoking Prevalence and Attributable Disease burden in 195 Countries and Territories, 1990-2015: a Systematic Analysis from the Global Burden of Disease Study 2015. Lancet (2017) 389:1885–906. 10.1016/S0140-6736(17)30819-X 28390697 PMC5439023

[2] Jha P Ramasundarahettige C Landsman V Rostron B Thun M Anderson RN 21st-Century Hazards of Smoking and Benefits of Cessation in the United States. N Engl J Med (2013) 368:341–50. 10.1056/NEJMsa1211128 23343063

[3] Aubin H-J Farley A Lycett D Lahmek P Aveyard P. Weight Gain in Smokers after Quitting Cigarettes: Meta-Analysis. Bmj (2012) 345:e4439. 10.1136/bmj.e4439 22782848 PMC3393785

[4] Tian J Venn A Otahal P Gall S. The Association between Quitting Smoking and Weight Gain: a Systemic Review and Meta-Analysis of Prospective Cohort Studies. Obes Rev (2015) 16:883–901. 10.1111/obr.12304 26114839

[5] Wang X Qin L-Q Arafa A Eshak ES Hu Y Dong J-Y. Smoking Cessation, Weight Gain, Cardiovascular Risk, and All-Cause Mortality: A Meta-Analysis. Nicotine Tob Res (2021) 23:1987–94. 10.1093/ntr/ntab076 33876246

[6] Yeh H-C Duncan BB Schmidt MI Wang NY Brancati FL. Smoking, Smoking Cessation, and Risk for Type 2 Diabetes Mellitus. Ann Intern Med (2010) 152:10–7. 10.7326/0003-4819-152-1-201001050-00005 20048267 PMC5726255

[7] Hur NW Kim HC Mo Nam C Ha Jee S Lee HC Suh I. Smoking Cessation and Risk of Type 2 Diabetes Mellitus: Korea Medical Insurance Corporation Study. Eur J Cardiovasc Prev Rehabil (2007) 14:244–9. 10.1097/01.hjr.0000239474.41379.79 17446803

[8] Pan A Wang Y Talaei M Hu FB Wu T. Relation of Active, Passive, and Quitting Smoking with Incident Type 2 Diabetes: a Systematic Review and Meta-Analysis. Lancet Diabetes Endocrinol (2015) 3:958–67. 10.1016/S2213-8587(15)00316-2 26388413 PMC4656094

[9] Hu Y Zong G Liu G Wang M Rosner B Pan A Smoking Cessation, Weight Change, Type 2 Diabetes, and Mortality. N Engl J Med (2018) 379:623–32. 10.1056/NEJMoa1803626 30110591 PMC6165582

[10] Sahle BW Chen W Rawal LB Renzaho AMN. Weight Gain after Smoking Cessation and Risk of Major Chronic Diseases and Mortality. JAMA Netw Open (2021) 4:e217044. 10.1001/jamanetworkopen.2021.7044 33904915 PMC8080225

[11] Choi JW Kim TH Han E. Smoking Cessation, Weight Change, Diabetes, and Hypertension in Korean Adults. Am J Prev Med (2021) 60:205–12. 10.1016/j.amepre.2020.08.024 33153837

[12] Zhao Y Hu Y Smith JP Strauss J Yang G. Cohort Profile: the China Health and Retirement Longitudinal Study (CHARLS). Int J Epidemiol (2014) 43:61–8. 10.1093/ije/dys203 23243115 PMC3937970

[13] Lian Y Yang L Gao M Jia C-X. Relationship of Frailty Markers and Socioeconomic Status to Incidence of Depressive Symptoms in a Community Cohort. J Am Med Directors Assoc (2021) 22:570–6. 10.1016/j.jamda.2020.08.026 33011096

[14] Kim BJ Kim BS Sung KC Kang JH Lee MH Park JR. Association of Smoking Status, Weight Change, and Incident Metabolic Syndrome in Men: a 3-year Follow-Up Study. Diabetes Care (2009) 32:1314–6. 10.2337/dc09-0060 19389815 PMC2699708

[15] Suwazono Y Dochi M Oishi M Tanaka K Morimoto H Sakata K. Longitudinal Effect of Smoking Cessation on Physical and Laboratory Findings. Am J Prev Med (2010) 38:192–200. 10.1016/j.amepre.2009.09.040 20117576

[16] Chia CW Egan JM Ferrucci L. Age-Related Changes in Glucose Metabolism, Hyperglycemia, and Cardiovascular Risk. Circ Res (2018) 123:886–904. 10.1161/CIRCRESAHA.118.312806 30355075 PMC6205735

[17] Stadler M Tomann L Storka A Wolzt M Peric S Bieglmayer C Effects of Smoking Cessation on β-cell Function, Insulin Sensitivity, Body Weight, and Appetite. Eur J Endocrinol (2014) 170:219–27. 10.1530/EJE-13-0590 24179100

[18] Balkau B Vierron E Vernay M Born C Arondel D Petrella A The Impact of 3-year Changes in Lifestyle Habits on Metabolic Syndrome Parameters: the D.E.S.I.R Study. Eur J Cardiovasc Prev Rehabil (2006) 13:334–40. 10.1097/01.hjr.0000214614.37232.f0 16926661 PMC4764669

[19] Kodama S Fujihara K Ishiguro H Horikawa C Ohara N Yachi Y Unstable Bodyweight and Incident Type 2 Diabetes Mellitus: A Meta-Analysis. J Diabetes Investig (2017) 8:501–9. 10.1111/jdi.12623 PMC5497032 28083921

[20] Park K-Y Hwang H-S Cho K-H Han K Nam GE Kim YH Body Weight Fluctuation as a Risk Factor for Type 2 Diabetes: Results from a Nationwide Cohort Study. J Clin Med (2019) 8:950. 10.3390/jcm8070950 PMC6678837 31261984

[21] Pani LN Nathan DM Grant RW. Clinical Predictors of Disease Progression and Medication Initiation in Untreated Patients with Type 2 Diabetes and A1C Less Than 7%. Diabetes Care (2008) 31:386–90. 10.2337/dc07-1934 18083790 PMC3829640

[22] Kataja-Tuomola M Sundell J Männistö S Virtanen MJ Kontto J Albanes D Short-term Weight Change and Fluctuation as Risk Factors for Type 2 Diabetes in Finnish Male Smokers. Eur J Epidemiol (2010) 25:333–9. 10.1007/s10654-010-9444-6 20352298

[23] Jung H-S Chang Y Eun Yun K Kim C-W Choi E-S Kwon M-J Impact of Body Mass index, Metabolic Health and Weight Change on Incident Diabetes in a Korean Population. Obesity (2014) 22:1880–7. 10.1002/oby.20751 24706434

[24] Lissner L Andres R Muller DC Shimokata H. Body Weight Variability in Men: Metabolic Rate, Health and Longevity. Int J Obes (1990) 14:373–83. 2361814

[25] Wallner SJ Luschnigg N Schnedl WJ Lahousen T Sudi K Crailsheim K Body Fat Distribution of Overweight Females with a History of Weight Cycling. Int J Obes (2004) 28:1143–8. 10.1038/sj.ijo.0802736 15263924

[26] Liu X Bragg F Yang L Kartsonaki C Guo Y Du H Smoking and Smoking Cessation in Relation to Risk of Diabetes in Chinese Men and Women: a 9-year Prospective Study of 0·5 Million People. Lancet Public Health (2018) 3:e167–e176. 10.1016/S2468-2667(18)30026-4 29548855 PMC5887081

[27] Oba S Noda M Waki K Nanri A Kato M Takahashi Y Smoking Cessation Increases Short-Term Risk of Type 2 Diabetes Irrespective of Weight Gain: the Japan Public Health Center-Based Prospective Study. PLoS One (2012) 7:e17061. 10.1371/journal.pone.0017061 22879858 PMC3409867

[28] Luo J Rossouw J Tong E Giovino GA Lee CC Chen C Smoking and Diabetes: Does the Increased Risk Ever Go Away? Am J Epidemiol (2013) 178:937–45. 10.1093/aje/kwt071 23817918 PMC3816526

